# Effects of Polymannuronic Acid on the Intestinal Microbiota in Mice after Long-Term Intragastric Administration

**DOI:** 10.3390/md22030125

**Published:** 2024-03-06

**Authors:** E Zhang, Qiang Wei, Xia Li, Shuliang Song

**Affiliations:** Marine College, Shandong University, Weihai 264209, China; 202020923@mail.sdu.edu.cn (E.Z.); wq18086420598@163.com (Q.W.)

**Keywords:** polymannuronic acid, intestinal flora, 16S rRNA gene sequencing, *Escherichia*, inflammatory factors

## Abstract

Polymannuronic acid (PM) is an alginate oligosaccharide derived from brown algae with a characterized structure and excellent biological activities. Herein, mice were given different doses of PM through 30-day-long-term intragastric administration, and the contents of the jejunum, ileum, and colon were analyzed by 16S rRNA gene sequencing technology for microbial diversity, and relevant experiments were verified according to the analysis results so as to comprehensively evaluate the effects of PM on the intestinal flora. The PM (400 mg/kg and 100 mg/kg) could regulate the microflora balance at the phylum level and increase the microflora richness in the jejunum, ileum, and colon of the mice. The PM could induce more strains that are negatively correlated with *Escherichia*, thereby reducing the relative abundance of *Escherichia*. Analysis of bacterial function showed that high and low doses of PM could promote lipid metabolism in the bacterial communities. Moreover, the PM could reduce serum total cholesterol and cholesterol ester levels in a concentration-dependent manner. High-dose PM could lead to colonic intestinal inflammation by increasing the relative abundance of multiple bacterial groups in the jejunum, ileum, and colon. Moreover, high-dose PM could increase lipopolysaccharide-binding protein and interleukin-1β levels. Therefore, the dose of PM plays an important role in its efficacy, and its biological activity is dosedifferent.

## 1. Introduction

**Figure 1:**
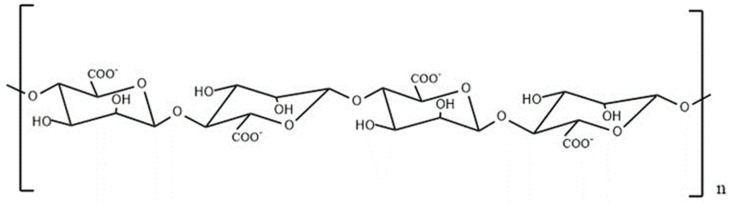
Chemical structure of polymannuronic acid.

Alginate is an anionic polymer commonly obtained from brown algae, such as kelp. Owing to its high biocompatibility, low toxicity, and relatively low cost, it has been widely used in food additives and biomedical fields [1,2]. However, the activity of alginate is limited due to its high molecular weight, long-chain structure, poor water solubility, and low bioavailability. Therefore, obtaining low-molecular-weight polysaccharides or oligosaccharides by degradation is of great significance in improving the bioavailability of polysaccharides. Polymannuronic acid (PM) is a brown alginate oligosaccharide derived from sodium alginate; its structure has been characterized (Figure 1). It has various biological activities such as antitumor, antioxidant, immuneregulation, obesityinhibition, blood pressurelowering, blood lipidlowering, and blood glucoselowering effects [3,4,5]. PM was prepared by acid hydrolysis, pH precipitation, ion exchange column chromatography, and gel column chromatography [6]. In PM, all M residues are connected by a β-1, 4-glucoside bond. The primary administration mode for the study of the pharmacological activity of PM is oral administration. However, most of the PM is retained in the gastrointestinal tract because of its low absorption rate. Whether the unabsorbed PM affects the intestinal flora and further affects the body has not been confirmed. In addition, from the perspective of long-term compliance in humans, oral administration of PM is considered the best route. Therefore, different doses of PM were administered to mice intragastrically to analyze and verify its long-term effects on the intestinal flora of mice using 16S rRNA gene sequencing of the jejunum, ileum, and colon contents. This study comprehensively evaluates the impact of the long-term oral administration of PM on mice, provides insights into the pharmacological activity of PM, and serves as an experimental basis for further research and clinical application of PM in humans.

## 2. Results

### 2.1. Effects of PM Intragastric Administration on Body Weight and Fecal Water Content in Mice

**Figure 2:**
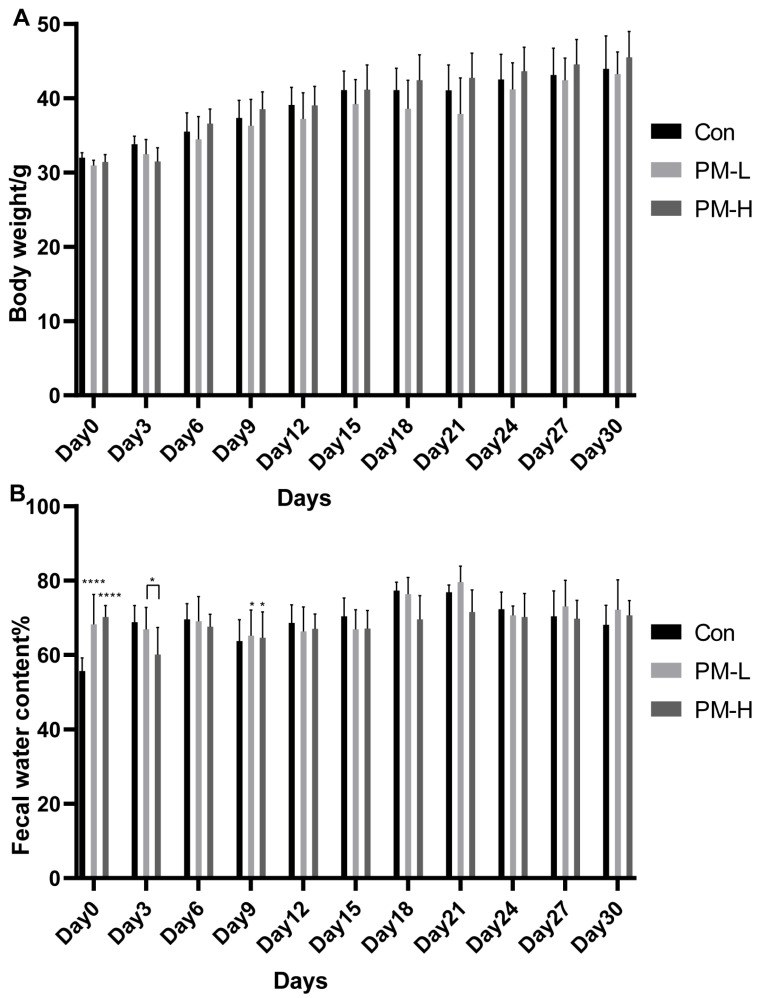
Body weight and fecal water content change during PM administration (*n* = 6). (**A**) Body weight changes in mice; (**B**) Changes in fecal water content in experimental vs. control mice: * *p* < 0.05, **** *p* < 0.0001.

Both high and low doses of oral PM reduced the body weights of the mice during administration (30 days) versus those of mice in the control group; however, the difference was insignificant (Figure 2A). This finding showed that the dose of PM used in our study had little effect on the body weights of the mice.

During the administration period (30 days), changes in the fecal water content in mice were generally stable and between 60% and 70% of the average level (Figure 2B). At the time of sampling, the mice defecated normally; the time for defecation was not long and was relatively average, and there were no signs of constipation. The feces were about the size of rice grains;they were black, not thin and soft, and lacked apparent odor. These properties were consistent with the characteristics of feces from healthy mice. There were significant differences in the fecal water content of mice in the early stage, indicating that the administration of PM could increase the fecal water content of mice in the early stage. After 3–6 days of adaptation, there were no significant differences in the fecal water content among the groups of mice measured at the later stage. The measurement results across different days also tended to be stable, indicating to a certain extent that the long-term oral administration of PM had no significant effect on intestinal fecal water content in healthy mice.

### 2.2. Effects of PM Intragastric Administration on the Organ Indices of Mice

**Figure 3:**
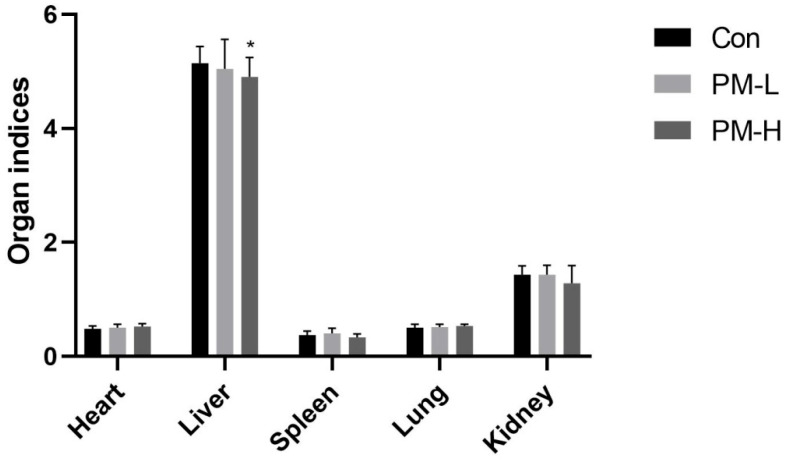
Effect of PM on the organ indices of mice after 30 days of intragastric administration (*n* = 6) vs. control: * *p* < 0.05.

After intragastric PM for 30 days, the appearance and color of the organs of mice were observed after dissection, and no signs of lesions were determined. The effect of PM on the organ indices of mice is shown in Figure 3. No significant differences in the heart, spleen, lung, and kidney indices were found in mice among all groups (*p* > 0.05); deviations within the group were small, indicating that the experimental dose of PM did not affect the heart, spleen, lung, and kidney indices of mice. The liver indices of mice in the PM-L and PM-H groups were slightly lower than those of mice in the control group, with the liver index of mice in the PM-H group being lower than that of mice in the PM-L group and significantly different compared with that of mice in the control group (*p* < 0.05).

### 2.3. 16S rRNA Gene Sequencing

Eighteen mice were randomly divided into a control group, PM-L (100 mg/kg), or PM-H group (400 mg/kg) with 6 mice per group, and PM was administered once daily. On the 30th day, the jejunum, ileum, and colon intestinal segments were separated under aseptic conditions, and the contents of each intestinal segment were taken in an aseptic EP tube. High-throughput 16S rRNA gene sequencing was performed for the above samples, and correlation analysis of the intestinal flora was performed.

#### 2.3.1. Analysis of Mouse Intestinal Flora with Operation Taxonomic Unit (OTU) Core-Pan Map

**Figure 4:**
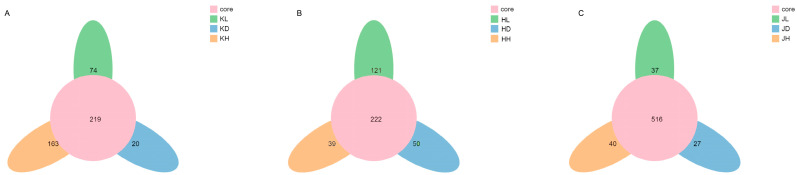
OTU petals of microflora in different groups of mice and in different intestinal segments of mice. (**A**) KD: jejunum control group; KL: jejunum low-dose group; KH: jejunum high-dose group; (**B**) HD: ileum control group; HL: ileum low-dose group; HH: ileum high-dose group; (**C**) JD: colon control group; JL: colon low-dose group; JH: colon high-dose group.

A petal diagram can show OTUs that are common or unique to all samples. The middle circle of the petal diagram indicates the number of OTUs in each experimental group, and the ellipse outside the middle circle indicates the number of OTUs unique to a group. Figure 4 shows that the number of common OTUs in the jejunum and ileum is relatively small, with 219 and 222 common OTUs, respectively, whereas the number of common OTUs in the colon is significantly increased, with 516 common OTUs. Based on the number of unique OTUs, the effects of long-term intragastric PM on the jejunum, ileum, and colon were gradually decreased and dose-dependent. Long-term PM administration had the most significant impact on jejunal microflora OTUs and the most negligible effect on colon microflora OTUs.

#### 2.3.2. Effect of PM on the Alpha Diversity of Intestinal Flora in Mice

Based on the rRNA data obtained from each sample, various diversity indices were analyzed to determine the effect of PM on the alpha diversity of intestinal flora in mice.

As seen in Table 1, Table 2 and Table 3, the coverage of samples in each group is close to 1, indicating that the results can truly reflect the findings in the samples. In the jejunum, ileum, and colon, the chao and ace indices in the low- and high-dose PM groups showed an increased trend compared with those in the control group, indicating that the long-term administration of PM could promote the increase in microflora abundance in the jejunum, ileum, and colon of mice. Except for the significant difference between the low-dose jejunum group and the control group (Table 1) with respect to the Shannon index and Simpson index, there was no significant difference between these indices in different intestinal segments and the control group after administration of other doses, indicating that the diversity of microflora in the jejunum, ileum, and colon of mice did not change much after PM intervention. Therefore, alpha diversity analysis revealed that different doses of PM could increase the abundance of microflora in the jejunum, ileum, and colon of mice, but different doses of PM had no significant effect on the diversity of microflora in the jejunum, ileum, and colon of mice.

#### 2.3.3. Effect of PM on the Beta Diversity of Intestinal Flora in Mice

**Figure 5:**
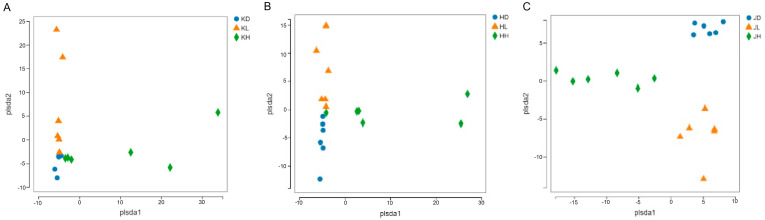
Beta diversity analysis of microbiota in the jejunum, ileum, and colon of mice after 30 days of intragastric administration of PM. (**A**) KD: jejunum control group; KL: jejunum low-dose group; KH: jejunum high-dose group; (**B**) HD: ileum control group; HL: ileum low-dose group; HH: ileum high-dose group; (**C**) JD: colon control group; JL: colon low-dose group; JH: colon high-dose group.

As seen in Figure 5, the microflora distribution in the jejunum, ileum, and colon of each group of mice was significantly different, indicating that different doses of PM had certain effects on the microflora structure in each intestinal segment of mice. The impact of low-dose PM on the microflora of all intestinal segments was consistent and distributed vertically along PLSDA2. Moreover, the impact of high-dose PM on the microflora of all intestinal segments was also consistent and distributed between the high-dose and control groups and distributed horizontally along PLSDA1.

#### 2.3.4. Analysis of Species Composition of the Microflora in Each Intestinal Segment after the Intragastric Administration of PM

**Figure 6:**
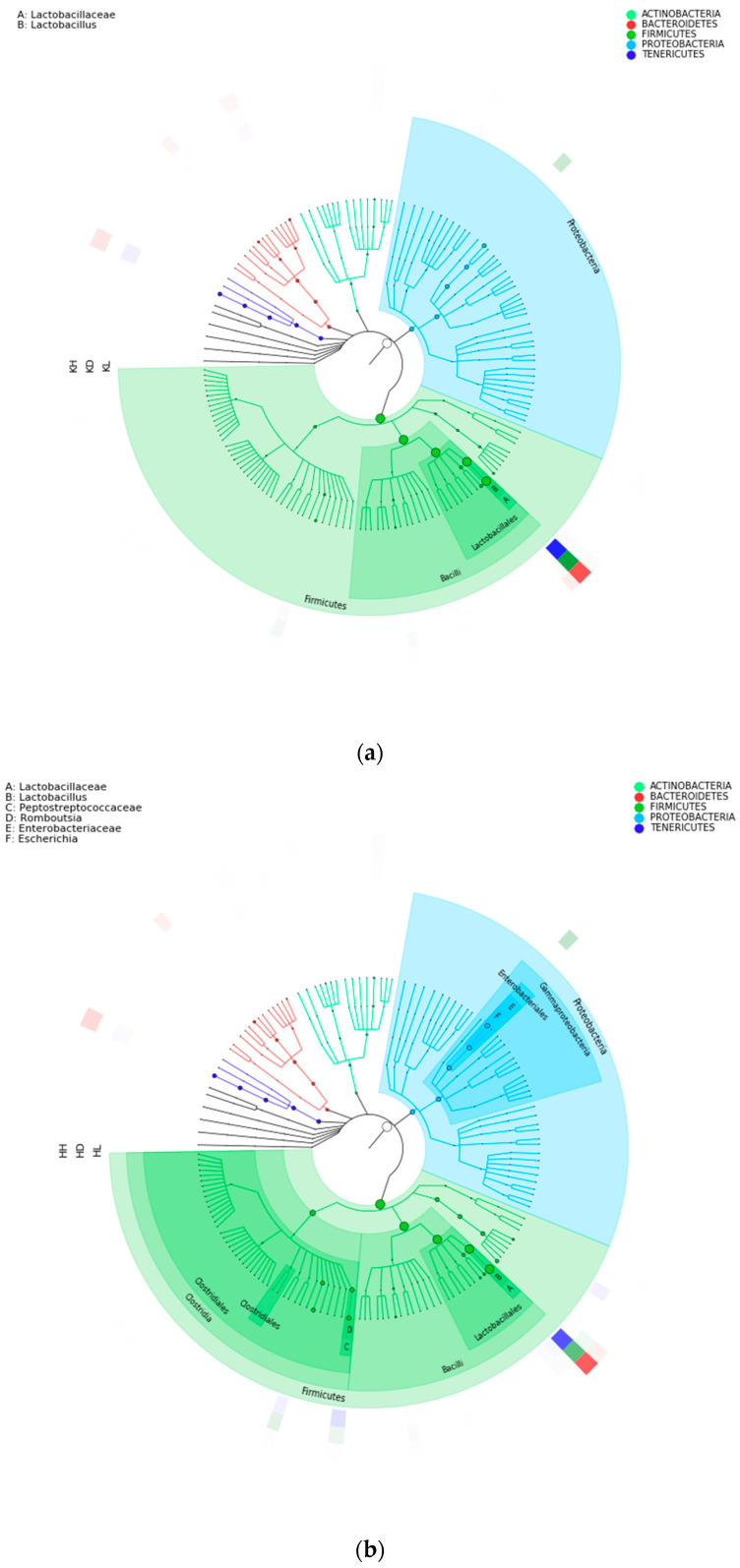

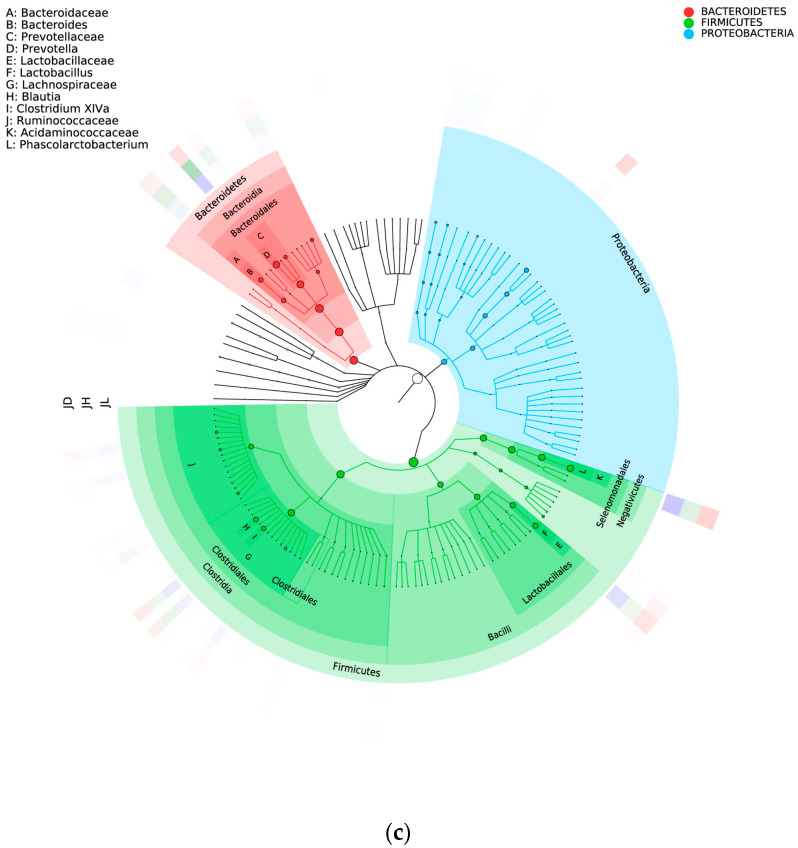
Species composition in each intestinal segment of mice after 30 days of intragastric administration of PM using GraPhlAn (version 1.1.3). Each circle of the evolutionary branch tree represents a hierarchy from the inside to the outside of the phylum, class, order, family, and genus level. The larger the number of nodes in a branching tree, the greater the relative abundance of the species. The outer ring is the relative abundance heat map, and each ring is an experimental group. Each experimental group corresponds to a color, and the color shade varies with the relative abundance of species. (**a**) KD: jejunum control group; KL: jejunum low-dose group; KH: jejunum high-dose group; (**b**) HD: ileum control group; HL: ileum low-dose group; HH: ileum high-dose group; (**c**) JD: colon control group; JL: colon low-dose group; JH: colon high-dose group.

According to the 16S rRNA gene sequencing results, the GraPhlAn software (version 1.1.3) was used to visually display the species composition of the flora in the jejunum, ileum, and colon at different classification levels in each experimental group of mice. Figure 6 shows that the microflora composition in the jejunum and ileum is similar to that at the phylum level. They all contain the following five phyla: *Firmicutes*, *Bacteroidetes*, *Proteobacteria*, *Actinobacteria*, and *Tenericutes*. The dominant bacterial groups in both the jejunum and ileum are *Firmicutes→Bacilli→Lactobacillales→Lactobacillaceae→Lactobacillus*. *Lactobacillus* of *Firmicutes* is dominant in the jejunum and ileum.

The microflora of the colon, jejunum, and ileum were significantly different, although the flora of the colon is predominantly composed of *Firmicutes*, *Bacteroidetes*, *Proteobacteria*, and three other phyla. However, the relative abundance of the microflora in the colon showed more diversity, dominance, and balance. Although *Lactobacillus* was still the dominant bacterial group, its relative abundance significantly decreased. The predominant flora of the colon mainly included the following three groups:

1. *Bacteroidetes*→*Bacteroidia*→*Bacteroidales*→*Prevotellaceae*→*Prevotella*;
2. *Firmicutes*→*Negativicutes*→*Selenomonadales*→*Acidaminococcaceae*→*Phascolarctobacterium*;
3. *Firmicutes*→*Bacilli*→*Lactobacillales*→*Lactobacillaceae*→*Lactobacillus*.

#### 2.3.5. Analysis of the Differences in the Composition of Dominant Species at the Portal Level of Each Intestinal Segment after the Intragastric Administration of PM

The *Bacteroidetes* and *Firmicutes* ratio (B/F ratio) at the phylum level is a vital reference index to measure the balance of the flora, and it is generally considered to be negatively correlated with obesity and inflammation [7,8].Studies have found that genetically obese mice have a lower B/F ratio compared with that of thin mice, possibly because *Firmicutes* helps obese mice absorb more calories from the consumed diet, leading to obesity [9].The statistical analyses of the B/F values of each intestinal segment and each experimental group of mice in our study are shown in Table 4. The long-term intragastric administration of PM increased the B/F ratio in the jejunum, ileum, and colon in a dose-dependent manner. Therefore, based on the B/F ratio, it can be inferred that the high and low doses of intragastric PM could promote the balance of intestinal flora in mice.

**Figure 7:**
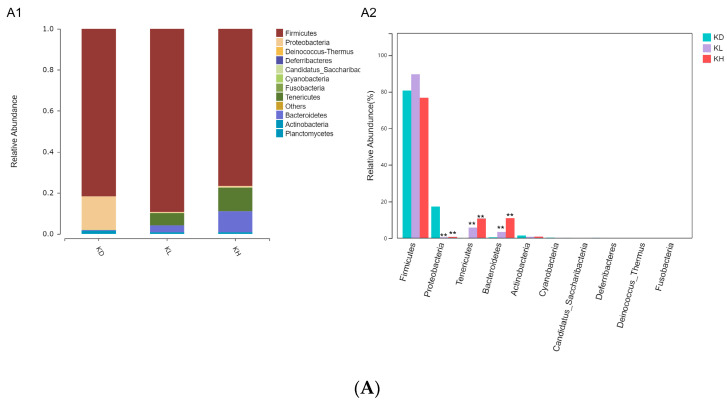

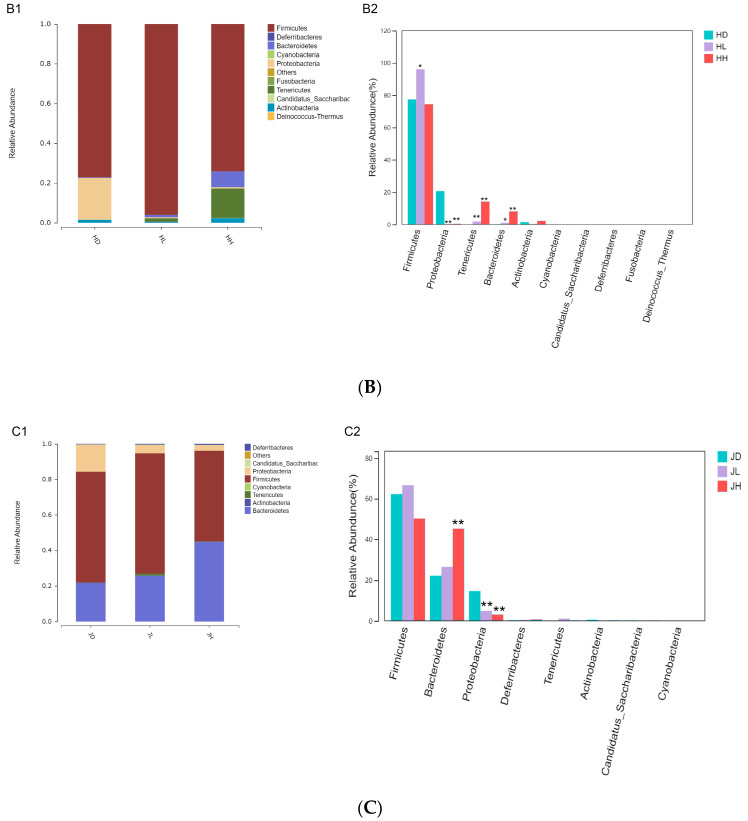
Differential analysis of dominant species at the phylum level of each intestinal segment in mice after 30 days of intragastric administration of PM. (**A**) KD: jejunum control group; KL: jejunum low-dose group; KH: jejunum high-dose group; (**A1**) relative abundance of jejunum microflora; (**A2**) difference analysis of jejunum microflora(relative abundance top 10); (**B**) HD: ileum control group; HL: ileum low-dose group; HH: ileum high-dose group; (**B1**) relative abundance of ileum microflora; (**B2**) difference analysis of ileum microflora(relative abundance top 10); (**C**) JD: colon control group; JL: colon low-dose group; JH: colon high-dose group; (**C1**) relative abundance of colon microflora; (**C2**) difference analysis of colon microflora(relative abundance top 10) vs. control: * *p* < 0.05, ** *p* < 0.01.

The species with the top 10 relative abundances were selected from each intestinal segment for phylum-level difference analysis using R (v3.4.1) software, and the results are shown in Figure 7. At the phylum level, the long-term intragastric administration of PM had a relatively consistent effect on the relative abundance of Proteobacteria flora in all intestinal segments. Administration of low- and high-dose PM significantly reduced *Proteobacteria*’s relative abundance in the jejunum, ileum, and colon. The effects of the long-term intragastric administration of PM on the jejunal and ileal microflora were similar, and the relative abundance of *Tenericutes* and *Bacteroidetes* increased significantly in mice administered both low- and high-dose PM. Mice in the low-dose group showed a significant increase in the relative abundance of *Firmicutes*(Figure 7B), whereas those in the high-dose group exhibited an increase in *Bacteroidetes* in the colon (Figure 7C).

#### 2.3.6. Analysis of the Horizontal Dominant Species Composition in Each Intestinal Segment after the Intragastric Administration of PM

At the subordinate level, the relative abundance of *Escherichia* microflora in the top 10 species in the jejunum, ileum, and colon was significantly reduced in both the low-dose and high-dose groups, and the relative abundance of *Prevotella* in each intestinal segment increased dramatically in the high-dose group compared with that in the control group. The effect of PM on the relative abundance of other species was significantly different in different intestinal segments.

**Figure 8:**
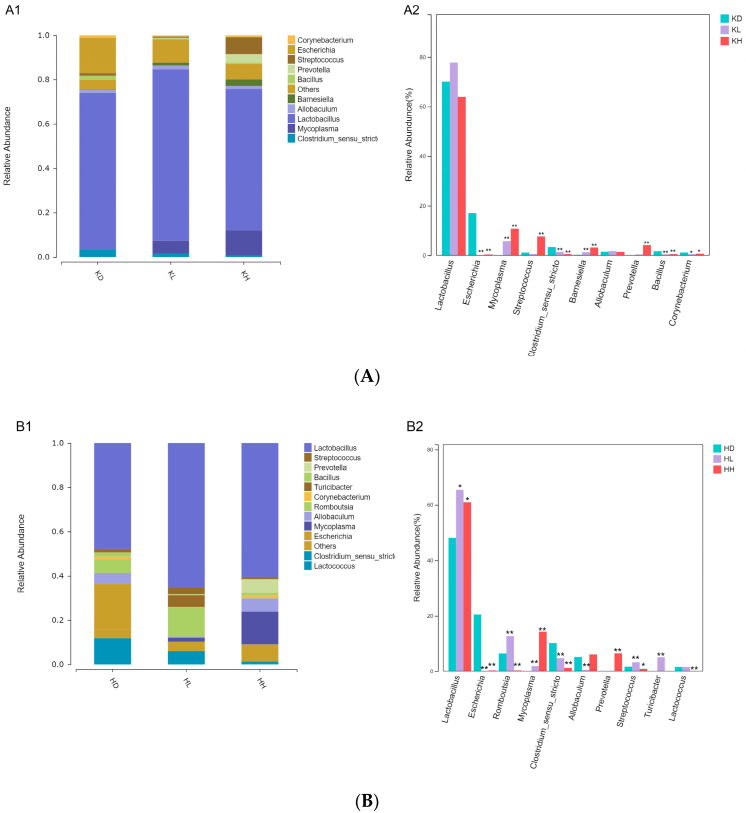

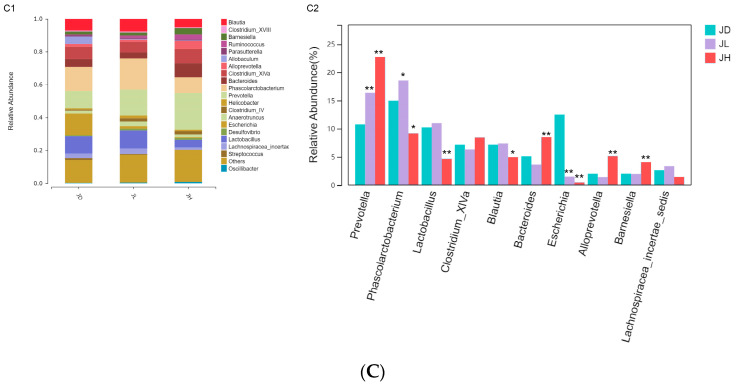
Differential analysis of the dominant species in each intestinal segment of mice at the genus level after 30 days of the intragastric administration of PM. (**A**) KD: jejunum control group; KL: jejunum low-dose group; KH: jejunum high-dose group; (**A1**) relative abundance of jejunum microflora; (**A2**) difference analysis of jejunum microflora(relative abundance top 10); (**B**) HD: ileum control group; HL: ileum low-dose group; HH: ileum high-dose group; (**B1**) relative abundance of ileum microflora; (**B2**) difference analysis of ileum microflora(relative abundance top 10); (**C**) JD: colon control group; JL: colon low-dose group; JH: colon high-dose group; (**C1**) relative abundance of colon microflora; (**C2**) difference analysis of colon microflora(relative abundance top 10) vs. control: * *p* < 0.05, ** *p* < 0.01.

Both high and low PM doses could significantly reduce the relative abundance of *Clostridiumsensustricto*, *Bacillus*, and *Corynebacterium* in the jejunum. The abundance of *Mycoplasma*, *Barnesiella*, and *Streptococcus* increased considerably in mice administered a high dose of PM (Figure 8A).

In the ileum, high and low PM doses reduced the relative abundance of *Clostridiumsensustricto* and significantly increased that of *Lactobacillus* and *Mycoplasma*. The relative abundances of *Romboutsia* and *Streptococcus* were different. Low doses of PM increased the relative abundances of these two genera, whereas high doses of PM decreased the relative abundances of these two genera. Low-dose PM could reduce the relative abundance of *Allobaculum* and significantly increase that of *Turicibacter*, whereas high-dose PM could significantly improve and decrease the relative abundance of *Lactococcus* (Figure 8B).

In the colon, high and low doses of PM increased the relative abundance of *Prevotella* compared with that in the control group. The low dose of PM tended to increase the abundance of *Phascolarctobacterium*, and the high dose of PM decreased the relative abundance of *Phascolarctobacterium*, *Lactobacillus*, and *Blautia*. An increase in the relative abundance of *Bacteroides*, *Alloprevotella*, and *Barnesiella* was noted (Figure 8C).

#### 2.3.7. Correlation Analysis of Dominant Species in Each Intestinal Segment after Intragastric Administration of PM

**Figure 9:**
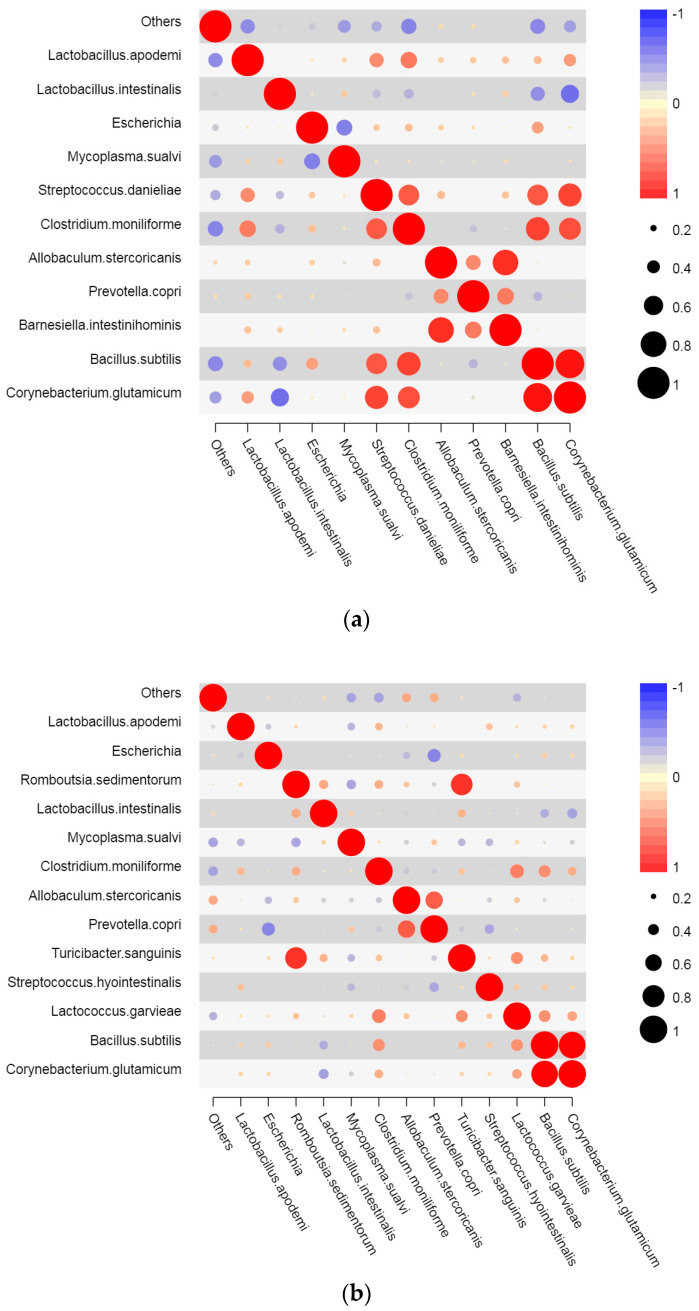

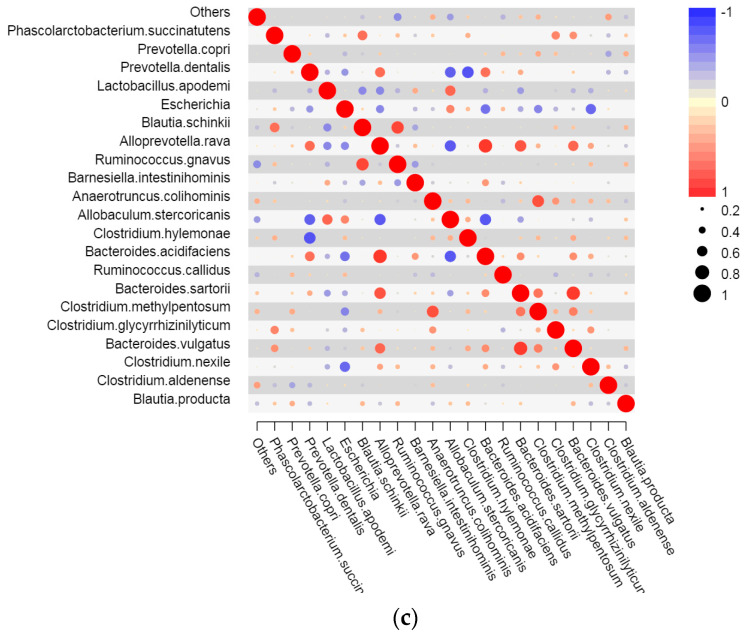
Heat map of correlation coefficients of microflora in each intestinal segment of mice after 30 days of intragastric administration of PM. (**a**): jejunum; (**b**): ileum; (**c**): colon.

R software (v3.4.1) was used to map Spearman correlation heat maps among the dominant species in the jejunum, ileum, and colon of the mice (Figure 9). The relative abundance of *Escherichia* in the jejunum decreased significantly, mainly due to the significant increase in the relative abundance of *Mycoplasma sualvi* (correlation coefficient with *Escherichia*: −0.496; Figure 9). However, *Bacillus subtilis* (correlation coefficient 0.375 with *Escherichia*), *Streptococcus daniele* (correlation coefficient 0.211 with *Escherichia*), *Clostridium moniliform* (correlation coefficient 0.249 with *Escherichia*), and *Allobaculumstercoricanis* (correlation coefficient 0.184 with *Escherichia*) were positively correlated with *Escherichia*. However, their relative abundance was low and also reduced (Figure 9a).

The relative abundance of *Escherichia* in the ileum decreased significantly, mainly due to *Prevotellacopri* (correlation coefficient with *Escherichia* was −0.476). In addition, *A.stercoricanis* (correlation coefficient −0.272 with *Escherichia*) and *Lactobacillus apodemi* (correlation coefficient −0.216 with *Escherichia*) also had a certain inhibitory effect on *Escherichia* (Figure 9b).

The interaction of the microflora in the colon is more complex than that in the jejunum and ileum, and the bacterial species are more diverse. As seen in Figure 9c, the relative abundance of *E. coli* decreased significantly, mainly due to *Bacteroides acidifies* (with a correlation coefficient of −0.556 with *Escherichia*), *Alloprevotellarava* (correlation coefficient −0.470 with *Escherichia*), *Clostridium methylpentosum* (correlation coefficient −0.482 with *Escherichia*), *Clostridium nexile* (correlation coefficient −0.593 with *Escherichia*), and *Prevotella*. Several bacteria, such as *Corynebacteriumdentalis* (correlation coefficient with *Escherichia* was −0.414), had a negative correlation, and only a few species, including *Allobaculumstercoricanis*,had a positive correlation with *C.dentalis* (Figure 9c).

The above analysis shows that after the long-term administration of PM, different intestinal segments have different negative correlations with *Escherichia*. In the jejunum, *M. sualvi* mainly had an inhibitory effect on *Escherichia*, and the relative abundance of positively related bacteria also led to the decline of *Escherichia*. In the ileum, the number of bacteria negatively associated with *Escherichia* increased, mainly *P.copri*, and the number of bacteria positively associated with *Escherichia* decreased. In the colon, the flora negatively associated with *Escherichia* increased further. Therefore, from the correlation point of view, the interaction of the intestinal flora was relatively healthy after 30 days of intragastric PM, indicating the effective inhibition of conditioned pathogen bacteria.

#### 2.3.8. Analysis of the Kyoto Encyclopedia of Genes and Genomes (KEGG) Function Difference in Each Intestinal Segment after Intragastric Administration of PM

**Figure 10:**
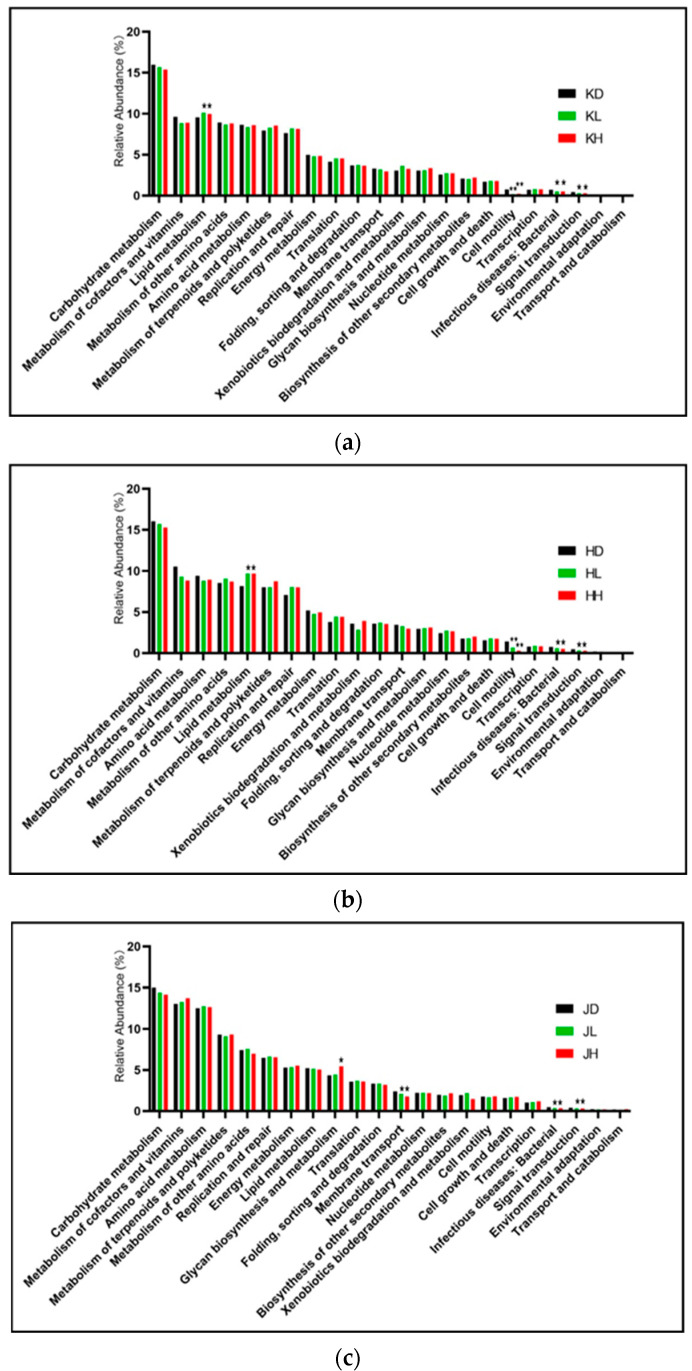
KEGG functional difference analysis of microflora in each intestinal segment of mice after 30 days of intragastric administration of PM. (**a**) KD: jejunum control group; KL: jejunum low-dose group; KH: jejunum high-dose group; (**b**) HD: ileum control group; HL: ileum low-dose group; HH: ileum high-dose group; (**c**) JD: colon control group; JL: colon low-dose group; JH: colon high-dose group vs. control: * *p* < 0.05, ** *p* < 0.01.

R software (v3.4.1) was used to analyze the KEGG function differences of the jejunum, ileum, and colon flora. Figure 10 shows that the main functions of the flora in each intestinal segment were similar. Carbohydrate metabolism and the metabolism of cofactors and vitamins were the top two factors, but there were no differences in these effects after PM administration. Long-term administration of PM intragastrically could significantly reduce the functions of infectious diseases (bacterial) and signal transduction in all intestinal segments. Among them, the results of reducing infectious diseases (bacterial) were consistent with the correlation analysis of dominant species in each intestinal segment, and the relative abundance of the harmful bacterium *Escherichia* was significantly reduced in each intestinal segment. However, the decrease in signal transduction function may affect related inflammatory factors, which requires further experimental verification.

High and low doses of PM similarly affected the functions of the jejunal and ileal microbiota. They could promote the function of lipid metabolism in the jejunum and ileum, and lipid metabolism was the leading effect. Long-term administration of PM may affect the lipid levels of mice; therefore, it is necessary to determine the blood lipid index. In addition, functions such as cell motility, infectious diseases (bacterial), and signal transduction decreased in both high- and low-dose groups (Figure 10a,c).

High-dose and low-dose PM had different effects on the colonic flora; the high dose of PM could significantly promote glycan biosynthesis and metabolism. High- and low-dose PM reduced membrane transport, infectious diseases (bacterial), and signal transduction functions (Figure 10c).

### 2.4. Validation of 16S rRNA Gene Analysis of Intestinal Flora in Mice

The liver index was reduced after the long-term intragastric administration of PM (Figure 3). 16S rRNA gene sequencing results showed that, in addition to the changes in colonic flora, the jejunal, ileal, and fecal flora at different times indicated the promotion of lipid metabolism after PM administration based on KEGG function difference analysis.Therefore, TC, FC, and cholesterol ester levels in the serum of mice were determined.

**Figure 11:**
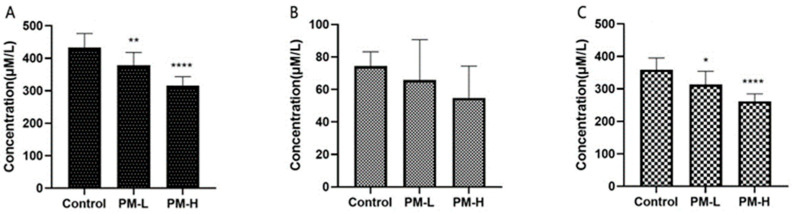
Serum cholesterol levels in mice after 30 days of intragastric administration of PM (*n* = 6). (**A**) Total cholesterol level; (**B**) Free cholesterol level; (**C**) Cholesterol ester level vs. control: * *p* < 0.05, ** *p* < 0.01, **** *p* < 0.001.

After 30 days of administration of PM, the TC content, FC content, and cholesterol ester content in the serum of each group of mice are shown in Figure 11. The long-term administration of oral PM could indeed reduce serum TC and cholesterol ester levels in a concentration-dependent manner; however, there was no significant difference in FC levels. The distribution of PM in vivo in tissues showed that PM could target the liver [6], indicating that it could reduce serum cholesterol levels by targeting the liver, thereby improving lipid metabolism in the liver and promoting the function of lipid metabolism by the intestinal flora. This result also indicated that PM has the potential to alleviate fatty liver and highlighted its benefit in improving liver health. Thus, it is necessary to further explore the lipid-lowering effect of PM in the later stages.

By increasing the relative abundance of Prevotella in the jejunum, ileum, colon, and stools, the high-dose intragastric PM decreased the relative abundance of the beneficial ileal bacteria *Romboutsia* and *Streptococcus*. The relative abundance of the beneficial bacteria *Phascolarctobacterium*, *Lactobacillus*, and *Blautia* in the colon decreased, and the relative abundance of the beneficial bacterium Bacteroides in the colon also decreased. This leads to a risk of inflammation of the colon and intestines. The low dose of PM decreased the abundance of *Allobaculum*, which is responsible for inflammation of the ileum, and increased the relative abundance of the beneficial ileal bacteria *Romboutsia* and *Streptococcus*. The increased relative abundance of beneficial bacteria, *Phascolarctobacterium* and *Blautia*, in the colon has the effect of reducing the levels of inflammatory cytokines. In addition, KEGG function difference analysis of fecal bacteria in different intestinal segments and at different times indicated that PM could reduce activity and signal transduction. Therefore, it is essential to further study the levels of LBP and interleukin (IL)-1β, the landmark inflammatory factors, in mouse serum.

**Figure 12:**
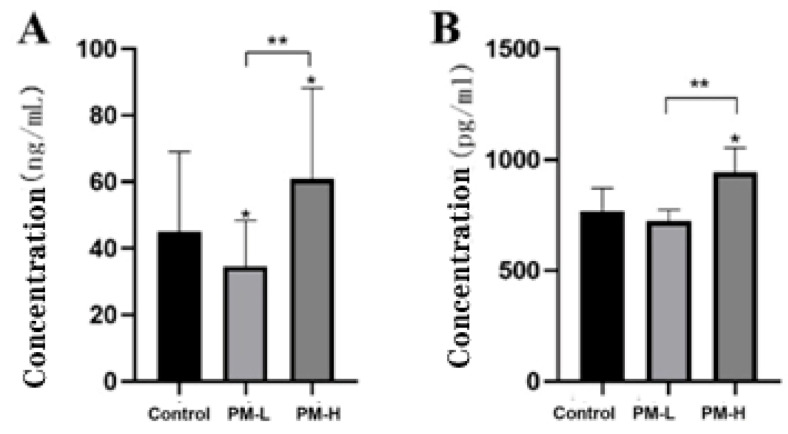
Levels of inflammatory factors in the serum of mice after 30 days of intragastric administration of PM (*n* = 6). (**A**) LBP levels; (**B**) IL-1β levels vs. control: * *p* < 0.05; PM-H vs. PM-L: ** *p* < 0.01.

Lipopolysaccharide-binding protein (LBP) is a 60 kDa lipid/phospholipid-binding and transfer proteinthat is mainly synthesized by liver cells and secreted into the blood [10].It belongs to type I acute phase reactive protein, which has a high affinity with the lipid A part of lipopolysaccharide and mediates the immune response triggered by lipopolysaccharide [11,12].Figure 12A shows that the long-term administration of low-dose PM by intragastric can reduce serum LBP levels in mice (*p* < 0.05), but the high-dose of PM can increase serum LBP levels in mice. There is a significant difference in serum LBP levels between the high-dose and low-dose groups after PM administration, which is consistent with the inferred results. Therefore, an appropriate PM dose is a more reasonable administration approach than a higher dose.

IL-1β is a potent pro-inflammatory cytokine essential for host defense against infections and injuries. It plays a role in many cellular activities, including cell proliferation, differentiation, and apoptosis [13]. IL-1β is the most characterized and studied of the 11 IL-1 family members [14]. Elevated IL-1β expression can produce numerous autoinflammatory syndromes and exacerbate injury during chronic diseases and acute tissue injury [15]. Figure 12B shows that long-term low-dose intragastric PM had no effect on serum IL-1β levels in mice; however, serum IL-1β levels in mice in the high-dose PM group were significantly higher than those of mice in the control group (*p* < 0.05). The serum IL-1β levels of mice in the low-dose and high-dose groups were significantly different (*p* < 0.01), consistent with the inferred results. Therefore, high-dose PM perfusion may promote the secretion of intestinal inflammatory factors.

## 3. Discussion

PM is a nondigestible oligosaccharide (NDO) characterized by its ability to resist stomach acid and degrading enzymes in mammals; however, it may be partially or completely degraded by certain gut microbes. Many studies have shown that the increased intake of NDOs may increase the relative abundance of some gut microbes, which is associated with a lower risk of obesity [16,17]. It has also been reported that NDOs have health benefits such as improving defecation, reducing appetite and postprandial blood glucose levels, regulating lipid metabolism, and promoting mineral absorption [18]. As a prebiotic, NDOs have attracted increasing attention and interest, which has helped improve the composition and metabolism of intestinal microbes and thus enhance intestinal health [19,20].

Owing to their unique physiological functions and important roles, gut microbes are considered “forgotten organs”. The gut ecosystem undergoes temporary changes throughout the life process with changes in various factors, which, in some cases, may lead to the destruction of the microbe–host symbiotic ecological balance [21]. The intestinal ecosystem plays a crucial role in maintaining host physiology; therefore, any disturbance can lead to a wide range of physiological disorders, including low inflammation, metabolic disorders, excessive fat accumulation, and loss of insulin sensitivity, thus increasing the risk of metabolic diseases [22]. Current scientific research focuses on understanding the mechanistic basis of the interaction between the gut microbiome and host metabolism in the occurrence and maintenance of host diseases and reveals the importance of the gut microbial–host–immune axis [23]. A few studies have shown that the relative abundance and composition of bacteria in the small intestine of different organisms are very different, and the dynamic change in bacterial diversity in the small intestine is much greater than that in the colon [24]. Colon microflora is primarily driven by the efficient degradation of complex nondigestible carbohydrates in the body. Still, the microflora of the small intestine is characterized by its ability to rapidly import and convert relatively small carbohydrates and its rapid adaptation to the overall nutrient supply [23]. Most studies focus on the colon microbiota, as this is where the greatest density and number of bacteria are found; most data are obtained from stool samples, followed by mucosal biopsies. While it is relatively easy to obtain fresh stool samples, the information obtained from these samples is not representative of the complete picture of the gut, particularly the community structure of microorganisms that colonize the surface of the intestinal walls. Therefore, in this study, the effects of PM on the microflora in different intestinal segments, including the jejunum, ileum, and colon, were analyzed.

Studies have shown that *Bacteroidetes* and *Firmicutes* dominate the human gut and participate in important physiological functions in the host gut. For example, some members of *Firmicutes* can metabolize dietary fiber in the colon into short-chain fatty acids (SCFAs) such as butyrate, which can provide 5–10% of energy to the body and exert anti-inflammatory effects and may be associated with obesity and diabetes [25]. In contrast, most members of *Bacteroidetes* are involved in carbohydrate metabolism and accomplish this process by expressing enzymes such as glycosyltransferase, glycoside hydrolase, and polysaccharide hydrolase and lyase, thereby playing a role in the normal development of the gut [26]. The B/F ratio has been used as an important indicator to evaluate the status of the human intestinal microbiota. It is significantly correlated with the human intestinal microbiota composition [27]. Similar findings have been reported in studies involving humans. For example, children who are obese have lower levels of *Bacteroidetes*, higher levels of *Firmicutes*, and lower levels of SCFAs in their intestines [28]. In addition, the microbial diversity in the guts of obese individuals is lower than that in the guts of lean individuals [29]. However, relevant studies have also shown that the decline in the B/F ratio with respect to obesity is still controversial [30,31].

After 30 days of PM administration, the structural composition of the microflora in the jejunum, ileum, and colon of mice in each group showed a significant increasing trend with respect to the B/F ratio in the low- and high-dose groups, and the increase in the high-dose group was more prominent. Therefore, long-term intragastric PM can significantly increase the B/F value of intestinal flora in the jejunum, ileum, colon, and other intestinal segments at the phylum level. This finding indicated that after PM entered the intestine, it could significantly affect the relative abundance and diversity of the intestinal flora, starting from the jejunum. This may be because the PM, as an important influencing factor or a carbon source, affected the relative abundance or diversity of the flora.

At the subordinate level, the long-term intragastric administration of PM significantly reduced the relative abundance of *Escherichia* in the jejunum, ileum, and colon, and the effect was obvious and consistent. KEGG function analysis of samples from different intestinal segments showed that the function of infectious diseases (bacterial) inthe intestinal flora was significantly reduced. Results from multiple analyses showed consistency. The bacteria in *Enterobacteriaceae* include many pathogenic bacteria genera, such as *E. coli*, *Salmonella*, and *Shigella* [32]. In a report that examined the bacterial flora of inflammatory bowel disease (IBD), individuals with a high risk of IBD were found to have high levels of inflammation and a high abundance of *Enterobacteriaceae* [33]. The genus *Escherichia* consists of five species, of which *E. coli* is the most popular [34]. There are many variants and different serotypes of *E. coli*, including symbiotic strains, and pathogenic strains that cause several human diseases and result in >2 million deaths annually [35]. Therefore, inhibiting the relative abundance of Escherichia by PM benefits intestinal microflora health and balance.

The relative abundance of *Prevotella* in each intestinal segment increased significantly in the high-dose PM group. The genus *Prevotella* is often considered a bacterium associated with a healthy plant-based diet, acting as a “probiotic” in the human body. It helps in the breakdown of proteins and carbohydrates. A decline in the abundance of *Prevotella* is often associated with certain diseases. *Prevotella* has the enzymes and gene clusters necessary for the fermentation and utilization of complex polysaccharides. A high-fiber diet can promote the intestinal type dominated by *Prevotella* [36], and excessive *Prevotella* may interact with other bacteria to induce visceral hypersensitivity by promoting carbohydrate fermentation and aggravating the symptoms of IBS [37]. DeVadder F. et al. colonized human *Prevotella* into mice and found that mice exhibited improved glucose tolerance and liver glycogen reserve [38]. *Prevotella* also contains enzymes that play an important role in mucin degradation, possibly leading to increased intestinal permeability. Moreover, treatment with *Prevotella* can aggravate the development of colitis in mice. Thus, it can be concluded that *Prevotella* has a dual role in the host gut and only has a beneficial effect on the host when the relative abundance is at a certain threshold. Experimental verification of inflammatory factors revealed that high-dose and long-term intragastric PM led to a significant increase in the key inflammatory factors LBP and IL-1β in the serum, which was consistent with the analysis results.

Analysis of KEGG function differences in different intestinal segment flora showed that the long-term intragastric PM could promote lipid metabolism in the intestinal flora, except in the colon. These findings were consistent with reports that NDOs have health benefits such as improving bowel movement, reducing appetite and postprandial blood glucose response, and regulating lipid metabolism [18]. TC, FC, and cholesterol ester levels in the serum of each group of mice also confirmed the result that PM could reduce serum TC and cholesterol ester levels in a concentration-dependent manner. Moreover, the liver index as a physiological health indicator in mice was also reduced, further confirming our findings.

Our study was the first to determine the effects of long-term intragastric PM administration on microorganisms in different intestinal segments. At the subordinate level, in addition to the consistent effects on the relative abundance of *Escherichia* and *Prevotella*, long-term intragastric PM showed different results in different intestinal segments.

In the jejunum, both high and low doses of PM could significantly reduce the relative abundance of *Clostridiumsensustricto*, *Bacillus*, and *Corynebacterium*. There was a significant increase in the abundance of *Mycoplasma*, *Barnesiella*, and *Streptococcus* in the high-dose group. Most bacteria in *Corynebacterium* are conditional pathogens [39]; thus, there is an obvious benefit in reducing their relative abundance. However, *Clostridiumsensustricto* and *Bacillus* are both SCFA-producing bacteria, and a decrease in their relative abundance is not conducive to the balance of the jejunal flora. *Barnesiella*, an anaerobic bacterium belonging to the family *Purpuromonas* of *Bacteroideae*, is a key gut-protecting bacterium that helps remove harmful bacteria from the gut [40]. The relative abundance of *Streptococcus* increased significantly in the high-dose PM group. Most bacteria belonging to the *Streptococcus* genus are not only nonpathogenic but are also beneficial to the host, especially *S. thermophilus*, which plays a role in regulating intestinal flora and immune regulation. Studies have shown that *S. thermophilus*, as a beneficial bacterium in the human intestine, can maintain the microecological balance in the intestine, inhibit spoilage bacteria, and improve digestibility [41]. The low dose of PM exerted a moderate regulatory effect on the composition of intestinal flora in the jejunal segment versus the high dose of PM, and a significant regulatory effect on the growth of related probiotics and pathogenic bacteria was observed.

Both high and low doses of PM reduced the relative abundance of the *Clostridiumsensustricto* microflora in the ileum and significantly increased the relative abundance of *Lactobacillus* and *Mycoplasma*. The relative abundances of *Romboutsia* and *Streptococcus* were different. The low dose of PM increased the relative abundances of these two genera, whereas high doses of PM decreased their relative abundances. The low dose of PM could decrease the relative abundance of *Allobaculum* and significantly increase that of *Turicibacter*, whereas the high dose of PM could significantly increase the relative abundance of *Lactococcus*. Most *Clostridium* species do not cause disease; therefore, a decrease in their relative abundance is generally beneficial to the intestinal flora balance. Intestinal *Lactobacillus* improves the digestion and absorption of carbohydrates, produces a variety of vitamins, inhibits the reproduction of spoilage bacteria and pathogenic bacteria in the intestine, reduces cholesterol levels in the blood, and maintains the balance of the intestinal flora [42]. *Mycoplasma* species rarely infect the gastrointestinal tract of patients and seldom cause intestinal diseases. Most studies have shown that *Rombuzia* is beneficial to humans and can reduce the risk of infections [43], whereas most *Streptococcus* species are nonpathogenic and normal bacteria. Therefore, the effect of low-dose PM is better than that of high-dose PM. *Allobaculum* can cause enteritis and is a key bacterial genus responsible for the imbalance in the intestinal flora [44]. Thus, the inhibitory effect of low-dose PM is better than that of high-dose PM. *Turicibacter* constitutes the beneficial intestinal flora and enhances the body’s immune function [45]. Therefore, in the ileal segment, the regulation of the ileal flora by low-dose PM mainly focused on the downregulation of pathogenic bacteria in various conditions, whereas high-dose PM upregulated the abundance of certain probiotic bacteria and pathogenic bacteria. Therefore, in general, the low dose of PM was more conducive to maintaining the microflora balance in the ileum of mice.

In the colon, the high and low doses of PM increased the relative abundance of *Prevotella* compared with that in the control group. The low-dose PM group showed a tendency for an increase in *Phascolarctobacterium*, whereas the high-dose PM group showed a tendency for a decrease in the relative abundance of *Phascolarctobacterium*, *Lactobacillus*, and *Blautia*. The relative abundance of *Bacteroides*, *Alloprevotella*, and *Barnesiella* increased. Koala produces SCFAs, including acetate and propionate, and may be related to the metabolic state and mood of the host, colonizing the human gastrointestinal tract in large numbers. The study found that individuals who lost weight more easily had higher levels of koala bacteria in their gut. In addition to its role in weight loss, this bacterium is a key regulatory factor in maintaining the dynamic balance of intestinal flora [46]. Therefore, an increase in the relative abundance of its flora may be beneficial. *Phascolarctobacterium*, *Lactobacillus*, and *Blautia* are beneficial bacteria. *Blautia* is an anaerobic bacteria with probiotic characteristicsthat prevent inflammation, promote SCFA production, and maintain intestinal homeostasis. It has potential probiotic properties [47];thus, the relative abundance of these three genera is not conducive to the balance of intestinal flora. The increase in *Bacteroides*, *Alloprevotella*, and *Barnesiella* had a certain beneficial effect. Therefore, in general, a low-dose intragastric of PM is more conducive to achieving a balance in the intestinal flora in the colon than a high-dose intragastric of PM.

Though differences in the analysis of bacterial KEGG function and lipid-related indicator detection showed that PM could promote lipid metabolism by the bacterial community and reduce serum TC and cholesterol esters in a concentration-dependent manner, high-dose PM (400 mg/kg) intragastric could lead to colonic intestinal inflammation by increasing the abundance of various bacteria in the jejunum, ileum, and colon. The measurement of the key inflammatory factors LBP and IL-1β in the serum has confirmed this hypothesis. Therefore, PM has the function of regulating some physiological activities of the body by regulating intestinal microorganisms, and the dose of PM has an important influence on its biological activity. The optimal dose of PM should be between 100 and 400 mg/kg, and the specific dose concentration needs to be determined by further animal experiments.

## 4. Materials and Methods

### 4.1. Main Experimental Instruments and Reagents

The main equipment used in this study was a pure water system (Elix5uv + Milli-QS, Merck Millipore, Boston, MA, USA), an oven (DHG-9143BS-Ⅲ, New Mio, Shanghai, China), an electronic balance (ME204, METTLER Toledo, Zurich, Switzerland), an ultra-low temperature refrigerator (DW-86L486, Haier, Qingdao, China), and an enzyme labeling instrument (Versa Max, Molecular Devices, Sunnyvale, CA, USA).

Main reagents: PM was prepared in our laboratory, and its average molecular weight was 16.83 kDa (the average degree of polymerization of PM was 95) [6]. The total cholesterol enzyme-assay kit and free cholesterol enzyme-assay kit were purchased from Pulilai, Beijing, China. A mouse lipopolysaccharide-binding protein (LBP) enzyme-linked immunosorbent assay (ELISA) kit was purchased from Wuhan Bude Wuhan, China. A mouse interleukin (IL)-1β ELISA kit was purchased from Biobiaceae Hangzhou, China. All other reagents were of analytically pure grade.

Experimental animals: specific-pathogen-free, 4-week-old male Kunming mice were selected and purchased from Qingdao DarenFucheng Animal Husbandry Co., Qingdao, China. We carried out the animal experiments in line with the ARRIVE guidelines and in accordance with the National Research Council’s Guide for the Care and Use of Laboratory Animals. All animal experiments were approved by the Medical Ethics Committee of Weihai Municipal Hospital (Weihai, China) protocol code was 2021084.

### 4.2. Grouping of Experimental Animals and PM Administration

The feeding conditions for mice were as follows: the air-conditioning temperature was set to 25 ± 1 °C, and the relative humidity was 50% ± 20%. Mice were subjected to a 12-h/12-h light/dark cycle. Throughout the experiment, mice were provided with free access to food. The drinking water was changed daily, the feed was supplemented daily, and the bedding was changed every 3 days. The first 7 days involved adaptive feeding; PM administration was commenced on the 8th day and recorded as day 0 of administration. All healthy mice were examined once daily during the experiment. Mice were randomly divided into three groups based on their body weight: the control group, the low-dose administration group (100 mg/kg, PM-L), and the high-dose administration group (400 mg/kg, PM-H), with 6 per group. Since there islittle literature on long-term intragastric administration of PM, we refer to the administration dose of fucoidan in our laboratory for PM administration [48].

Mice in each group were labeled and numbered, and those in the PM-L and PM-H groups were given intragastric administration of PM solution at the corresponding dose for 30 days, once a day at the same time. The PM concentration was adjusted according to their body weight. Mice in the control group were administered the same amount of normal saline intragastrically. Mice were provided access to food and water ad libitum during the experiment.

### 4.3. Monitoring the Changes in Body Weight and Fecal Water Content in Mice

Mice were weighed every 3 days, and their weights were monitored. The weights of each group of mice are shown as the mean ± standard deviation.

Monitoring of fecal water content: A certain amount of mouse feces was collected every 3 days, and the fecal water content of mice was determined using the dry weight loss method. The Eppendorf (EP) tube was numbered, and the empty EP tube (M1) was weighed using a 1/10,000 balance. After sampling fresh fecal samples, the mass (M2) was obtained, and the EP tube was put into the oven at 105 °C for drying. After drying, the weight (M3) was recorded when a constant reading was obtained. Formula (1) was used to calculate the fecal water content.

Water content of feces = 100% × (M2 − M3)/(M2 − M1)(1)

### 4.4. Sampling of the Blood, Intestinal Segments, and Organs of Mice

After 30 days of administration, blood was extracted from the eyeballs of mice under sterile conditions. Whole blood was placed at room temperature for 30 min to 2 h to avoid hemolysis caused by violent shaking. After natural coagulation of whole blood and precipitation of the serum, the samples were centrifuged at 1000–2000 rpm for 10 min at 4 °C. The yellow supernatant was separated to obtain the serum, packed, and frozen in an ultra-low-temperature refrigerator at −40 °C.

After extracting blood from the eyeballs of mice, their chest and abdomen were opened under sterile conditions, and each mouse’s heart, liver, spleen, lungs, and kidneys were removed. The fat and connective tissue were stripped away. The surface was cleaned with phosphate-buffered saline, wiped with absorbent paper, and weighed. The organ indices were calculated using Formula (2) [49]. Next, the jejunum, ileum, and colon were separated, and the contents of each intestinal segment were taken into a sterile EP tube and stored at −40 °C in an ultra-low temperature refrigerator.

Organ index = 100% × organ weight/total body weight(2)

### 4.5. Sample Collection and 16S rRNA Gene Sequencing to Determine Intestinal Flora

The contents of each intestinal segment (jejunum, ileum, and colon) of mice in the blank control group, PM-L group, and PM-H group obtained using the method stated in Section 2.4 were sent to Shenzhen BGI Gene Technology Service Co., Ltd., Shenzhen, China, for extraction of the total DNA of the microflora and high-throughput 16S rRNA gene sequencing in the V3–V4 region.

### 4.6. Verification of Findings from 16S rRNA Analysis of Mouse Intestinal Flora

Based on the results from 16S rRNA gene analysis of the intestinal flora of mice, the prepared serum (as described in Section 2.4) was further analyzed using a total cholesterol (TC) enzyme assay kit and a free cholesterol (FC) enzyme assay kit. TC and FC content in the serum were calculated using Formula (3) [50].

Cholesterol ester content (CE) = TC content − FC content(3)

Serum was prepared as stated in Section 2.4, and an ELISA kit was used to determine the levels of serum cytokines, including lipopolysaccharide-binding protein (LBP) and interleukin-1β (IL-1β).

### 4.7. Data Analysis

Except for 16S rRNA information data analysis, all other data from the groups are expressed as mean ± standard deviation. Data were processed using ordinary one-way analysis of variance in GraphPad Prism 8.0.1 to analyze whether there were significant differences between groups. Comparisons among multiple groups were performed using Tukey’s method.

## 5. Conclusions

Analysis of the intestinal microbial diversity showed that PM could regulate the microflora balance in the jejunum, ileum, and colon at the portal level. The microflora abundance in the jejunum, ileum, and colon increased after the administration of PM for 30 days. Analysis of the differences in the microflora in each intestinal segment and analysis of the flora correlation network diagram revealed that the long-term administration of PM could induce more strains and lead to a negative correlation with *Escherichia*, thereby reducing the abundance of *Escherichia*. Difference analysis of bacterial KEGG function showed that high and low doses of PM could promote lipid metabolism by the bacterial community and reduce serum TC and cholesterol esters in a concentration-dependent manner. Analysis of bacterial species composition and KEGG function showed that high-dose intragastric PM leads to colonic intestinal inflammation by increasing the abundance of various bacteria in the jejunum, ileum, and colon and increasing the levels of LBP and IL-1β.

## Figures and Tables

**Table 1:** Alpha diversity analysis of jejunal flora in each group of mice (*n* = 6).

Group	Chao	Ace	Shannon	Simpson	Coverage
control	153.83 ± 21.08	172.28 ± 17.90	1.69 ± 0.21	0.40 ± 0.16	0.9995 ± 0.0002
L-group	271.22 ± 51.09 **	275.77 ± 49.98 **	2.05 ± 0.47 *	0.39 ± 0.10	0.9992 ± 0.0003
H-group	306.19 ± 60.01 **	311.22 ± 49.91 **	1.55 ± 0.37	0.43 ± 0.10	0.9992 ± 0.0003

L-group: low-dose group; H-group: high-dose group vs. control: * *p* < 0.05, ** *p* < 0.01.

**Table 2:** Alpha diversity analysis of ileal flora in each group of mice (*n* = 6).

Group	Chao	Ace	Shannon	Simpson	Coverage
control	196.17 ± 59.35	165.14 ± 13.36	1.64 ± 0.31	0.36 ± 0.10	0.9993 ± 0.0002
L-group	211.21 ± 45.06	204.14 ± 40.38 *	1.68 ± 0.43	0.30 ± 0.13	0.9991 ± 0.0002
H-group	248.17 ± 51.95 *	237.57 ± 37.20 **	1.62 ± 0.43	0.40 ± 0.07	0.9990 ± 0.0003

L-group: low-dose group; H-group: high-dose group vs. control: * *p* < 0.05, ** *p* < 0.01.

**Table 3:** Alpha diversity analysis of colonic flora in each group of mice (*n* = 6).

Group	Chao	Ace	Shannon	Simpson	Coverage
control	373.19 ± 36.04	370.27 ± 40.11	3.54 ± 0.10	0.11 ± 0.04	0.9989 ± 0.0002
L-group	427.84 ± 38.70 *	430.34 ± 39.68 *	3.52 ± 0.16	0.11 ± 0.02	0.9998 ± 0.0002
H-group	455.38 ± 37.00 **	450.81 ± 40.94 **	3.42 ± 0.17	0.12 ± 0.02	0.9998 ± 0.0002

L-group: low-dose group; H-group: high-dose group vs. control: * *p* < 0.05, ** *p* < 0.01.

**Table 4:** Statistical analyses of B/F ratios in each intestinal segment after 30 days of intragastric administration of PM.

Group	Jejunum	Ileum	Colon
control	0.0040	0.0034	0.3561
L-group	0.0370 **	0.0110 **	0.3981 *
H-group	0.1330 **	0.1095 **	0.9001 **

L-group: low-dose group; H-group: high-dose group vs. control: * *p* < 0.05, ** *p* < 0.01.

## Data Availability

The datasets presented in this article are not readily available because the data are part of an ongoing study.

## References

[1] Wee S., Gombotz W.R. (1998). Protein release from alginate matrices. Adv. Drug Deliv. Rev..

[2] Remya R.R., Samrot A.V., Kumar S.S., Mohanavel V., Karthick A., Chinnaiyan V.K., Umapathy D., Muhibbullah M. (2022). Bioactive Potential of Brown Algae. Adsorpt. Sci. Technol..

[3] Xing M.C., Cao Q., Wang Y., Xiao H., Zhao J.R., Zhang Q., Ji A.G., Song S.L. (2020). Advances in Research on the Bioactivity of Alginate Oligosaccharides. Mar. Drugs.

[4] Zhang C., Li M., Rauf A., Khalil A.A., Shan Z., Chen C., Rengasamy K.R.R., Wan C. (2023). Process and applications of alginate oligosaccharides with emphasis on health beneficial perspectives. Crit. Rev. Food Sci. Nutr..

[5] Mrudulakumari Vasudevan U., Lee O.K., Lee E.Y. (2021). Alginate derived functional oligosaccharides: Recent developments, barriers, and future outlooks. Carbohydr. Polym..

[6] Song S., Wei Q., Wang K., Yang Q., Wang Y., Ji A., Chen G. (2022). Fluorescent Labeling of Polymannuronic Acid and Its Distribution in Mice by Tail Vein Injection. Mar. Drugs.

[7] Wang J.-L., Xiu C.-K., Yang J., Wang X., Hu Y.-H., Fang J.-Y., Lei Y. (2020). Effect of Ginseng Radix et Rhizoma, Notoginseng Radix et Rhizoma and Chuanxiong Rhizoma extracts on intestinal flora of vascular aging mice induced by high glucose and high lipid. Zhongguo Zhong Yao Za Zhi = Zhongguo Zhongyao Zazhi = China J. Chin. Mater. Med..

[8] Wei S., Bahl M.I., Baunwall S.M.D., Hvas C.L., Licht T.R. (2021). Determining Gut Microbial Dysbiosis: A Review of Applied Indexes for Assessment of Intestinal Microbiota Imbalances. Appl. Environ. Microbiol..

[9] Turnbaugh P.J., Ley R.E., Mahowald M.A., Magrini V., Mardis E.R., Gordon J.I. (2006). An obesity-associated gut microbiome with increased capacity for energy harvest. Nature.

[10] Weiss J. (2003). Bactericidal/permeability-increasing protein (BPI) and lipopolysaccharide-binding protein (LBP): Structure, function and regulation in host defence against Gram-negative bacteria. Biochem. Soc. Trans..

[11] Schumann R.R. (1992). Function of lipopolysaccharide (LPS)-binding protein (LBP) and CD14, the receptor for LPS/LBP complexes: A short review. Res. Immunol..

[12] Sakura T., Morioka T., Shioi A., Kakutani Y., Miki Y., Yamazaki Y., Motoyama K., Mori K., Fukumoto S., Shoji T. (2017). Lipopolysaccharide-binding protein is associated with arterial stiffness in patients with type 2 diabetes: A cross-sectional study. Cardiovasc. Diabetol..

[13] Dinarello C.A. (1996). Biologic basis for interleukin-1 in disease. Blood.

[14] Takeuchi O., Akira S. (2010). Pattern recognition receptors and inflammation. Cell.

[15] Lopez-Castejon G., Brough D. (2011). Understanding the mechanism of IL-1β secretion. Cytokine Growth Factor. Rev..

[16] Asadpoor M., Ithakisiou G.-N., Henricks P.A.J., Pieters R., Folkerts G., Braber S. (2021). Non-Digestible Oligosaccharides and Short Chain Fatty Acids as Therapeutic Targets against Enterotoxin-Producing Bacteria and Their Toxins. Toxins.

[17] Nie Q.X., Chen H.H., Hu J.L., Tan H.Z., Nie S.P., Xie M.Y., Doyle M.P., McClements D.J. (2020). Effects of Nondigestible Oligosaccharides on Obesity. Annual Review of Food Science and Technology.

[18] Liu Y., Chen J., Tan Q., Deng X., Tsai P.J., Chen P.H., Ye M., Guo J., Su Z. (2020). Nondigestible Oligosaccharides with Anti-Obesity Effects. J. Agric. Food Chem..

[19] Zmora N., Suez J., Elinav E. (2019). You are what you eat: Diet, health and the gut microbiota. Nat. Rev. Gastroenterol. Hepatol..

[20] Fernandes R., do Rosario V.A., Mocellin M.C., Kuntz M.G.F., Trindade E.B.S.M. (2017). Effects of inulin-type fructans, galacto-oligosaccharides and related synbiotics on inflammatory markers in adult patients with overweight or obesity: A systematic review. Clin. Nutr..

[21] Nazli A., Yang P.C., Jury J., Howe K., Watson J.L., Soderholm J.D., Sherman P.M., Perdue M.H., McKay D.M. (2004). Epithelia under metabolic stress perceive commensal bacteria as a threat. Am. J. Pathol..

[22] Boulange C.L., Neves A.L., Chilloux J., Nicholson J.K., Dumas M.-E. (2016). Impact of the gut microbiota on inflammation, obesity, and metabolic disease. Genome Med..

[23] Marchesi J.R., Adams D.H., Fava F., Hermes G.D.A., Hirschfield G.M., Hold G., Quraishi M.N., Kinross J., Smidt H., Tuohy K.M. (2016). The gut microbiota and host health: A new clinical frontier. Gut.

[24] Zoetendal E.G., Raes J., van den Bogert B., Arumugam M., Booijink C.C.G.M., Troost F.J., Bork P., Wels M., de Vos W.M., Kleerebezem M. (2012). The human small intestinal microbiota is driven by rapid uptake and conversion of simple carbohydrates. ISME J..

[25] Galvez-Ontiveros Y., Paez S., Monteagudo C., Rivas A. (2020). Endocrine Disruptors in Food: Impact on Gut Microbiota and Metabolic Diseases. Nutrients.

[26] Cantarel B.L., Lombard V., Henrissat B. (2012). Complex Carbohydrate Utilization by the Healthy Human Microbiome. PLoS ONE.

[27] dos Santos Pereira Indiani C.M., Rizzardi K.F., Castelo P.M., Caldas Ferraz L.F., Darrieux M., Parisotto T.M. (2018). Childhood Obesity and Firmicutes/Bacteroidetes Ratio in the Gut Microbiota: A Systematic Review. Child. Obes..

[28] Riva A., Borgo F., Lassandro C., Verduci E., Morace G., Borghi E., Berry D. (2017). Pediatric obesity is associated with an altered gut microbiota and discordant shifts in Firmicutes populations. Environ. Microbiol..

[29] Le Chatelier E., Nielsen T., Qin J., Prifti E., Hildebrand F., Falony G., Almeida M., Arumugam M., Batto J.-M., Kennedy S. (2013). Richness of human gut microbiome correlates with metabolic markers. Nature.

[30] Duncan S.H., Lobley G.E., Holtrop G., Ince J., Johnstone A.M., Louis P., Flint H.J. (2008). Human colonic microbiota associated with diet, obesity and weight loss. Int. J. Obes..

[31] Ley R.E., Turnbaugh P.J., Klein S., Gordon J.I. (2006). Microbial ecology—Human gut microbes associated with obesity. Nature.

[32] Walterson A.M., Stavrinides J. (2015). Pantoea: Insights into a highly versatile and diverse genus within the Enterobacteriaceae. Fems Microbiol. Rev..

[33] Metwaly A., Reitmeier S., Haller D. (2022). Microbiome risk profiles as biomarkers for inflammatory and metabolic disorders. Nat. Rev. Gastroenterol. Hepatol..

[34] Croxen M.A., Law R.J., Scholz R., Keeney K.M., Wlodarska M., Finlay B.B. (2013). Recent Advances in Understanding Enteric Pathogenic Escherichia coli. Clin. Microbiol. Rev..

[35] Kaper J.B., Nataro J.P., Mobley H.L.T. (2004). Pathogenic Escherichia coli. Nat. Rev. Microbiol..

[36] Tett A., Pasolli E., Masetti G., Ercolini D., Segata N. (2021). Prevotella diversity, niches and interactions with the human host. Nat. Rev. Microbiol..

[37] Ma W.J., Nguyen L.H., Song M.Y., Wang D.D., Franzosa E.A., Cao Y., Joshi A., Drew D.A., Mehta R., Ivey K.L. (2021). Dietary fiber intake, the gut microbiome, and chronic systemic inflammation in a cohort of adult men. Genome Med..

[38] De Vadder F., Kovatcheva-Datchary P., Zitoun C., Duchampt A., Backhed F., Mithieux G. (2016). Microbiota-Produced Succinate Improves Glucose Homeostasis via Intestinal Gluconeogenesis. Cell Metab..

[39] Pan L., Han P., Ma S., Peng R., Wang C., Kong W., Cong L., Fu J., Zhang Z., Yu H. (2020). Abnormal metabolism of gut microbiota reveals the possible molecular mechanism of nephropathy induced by hyperuricemia. Acta Pharm. Sin. B.

[40] Rinninella E., Cintoni M., Raoul P., Gasbarrini A., Mele M.C. (2020). Food Additives, Gut Microbiota, and Irritable Bowel Syndrome: A Hidden Track. Int. J. Environ. Res. Public Health.

[41] Vitetta L., Llewellyn H., Oldfield D. (2019). Gut Dysbiosis and the Intestinal Microbiome: Streptococcus thermophilus a Key Probiotic for Reducing Uremia. Microorganisms.

[42] Heeney D.D., Gareau M.G., Marco M.L. (2018). Intestinal Lactobacillus in health and disease, a driver or just along for the ride?. Curr. Opin. Biotech..

[43] Magruder M., Edusei E., Zhang L., Albakry S., Satlin M.J., Westblade L.F., Malha L., Sze C., Lubetzky M., Dadhania D.M. (2020). Gut commensal microbiota and decreased risk for Enterobacteriaceae bacteriuria and urinary tract infection. Gut Microbes.

[44] Li Q., Cui Y., Xu B., Wang Y., Lv F., Li Z., Li H., Chen X., Peng X., Chen Y. (2021). Main active components of Jiawei Gegen Qinlian decoction protects against ulcerative colitis under different dietary environments in a gut microbiota-dependent manner. Pharmacol. Res..

[45] Su J., Su L., Li D., Shuai O., Zhang Y., Liang H., Jiao C., Xu Z., Lai Y., Xie Y. (2018). Antitumor Activity of Extract From the Sporoderm-Breaking Spore of Ganoderma lucidum: Restoration on Exhausted Cytotoxic T Cell With Gut Microbiota Remodeling. Front. Immunol..

[46] Bhandarkar N.S., Mouatt P., Majzoub M.E., Thomas T., Brown L., Panchal S.K. (2021). Coffee Pulp, a By-Product of Coffee Production, Modulates Gut Microbiota and Improves Metabolic Syndrome in High-Carbohydrate, High-Fat Diet-Fed Rats. Pathogens.

[47] Liu X.M., Mao B.Y., Gu J.Y., Wu J.Y., Cui S.M., Wang G., Zhao J.X., Zhang H., Chen W. (2021). Blautia-a new functional genus with potential probiotic properties?. Gut Microbes.

[48] Wei Q., Xing M., Wang K., Yang Q., Zhao J., Wang Y., Li X., Ji K., Song S. (2022). Fucoidan Is Not Completely Dependent on Degradation to Fucose to Relieve Ulcerative Colitis. Pharmaceuticals.

[49] Yi R., Tan F., Zhou X., Mu J., Li L., Du X., Yang Z., Zhao X. (2020). Effects of Lactobacillus fermentum CQPC04 on Lipid Reduction in C57BL/6J Mice. Front. Microbiol..

[50] Wang Y., Gunewardena S., Li F., Matye D.J., Chen C., Chao X., Jung T., Zhang Y., Czerwiński M., Ni H.M. (2020). An FGF15/19-TFEB regulatory loop controls hepatic cholesterol and bile acid homeostasis. Nat. Commun..

